# Clinical features and treatment options for pediatric adrenal incidentalomas: a retrospective single center study

**DOI:** 10.1186/s12887-024-04673-7

**Published:** 2024-03-16

**Authors:** Xiaojiang Zhu, Saisai Liu, Yimin Yuan, Nannan Gu, Jintong Sha, Yunfei Guo, Yongji Deng

**Affiliations:** Department of Urology, Children’s Hospital of Nanjing Medical University, 72 Guangzhou Road, Nanjing, 210008 Jiangsu China

**Keywords:** Adrenal incidentaloma, Children, Retrospective, Clinical analysis

## Abstract

**Background:**

The aim of this study was to investigate the clinical features and treatment options for pediatric adrenal incidentalomas(AIs) to guide the diagnosis and treatment of these tumors.

**Methods:**

The clinical data of AI patients admitted to our hospital between December 2016 and December 2022 were collected and retrospectively analyzed. All patients were divided into neonatal and nonneonatal groups according to their age at the time of the initial consultation.

**Results:**

In the neonatal group, 13 patients were observed and followed up, and the masses completely disappeared in 8 patients and were significantly reduced in size in 5 patients compared with the previous findings. Four patients ultimately underwent surgery, and the postoperative pathological diagnosis was neuroblastoma in three patients and teratoma in one patient. In the nonneonatal group, there were 18 cases of benign tumors, including 9 cases of ganglioneuroma, 2 cases of adrenocortical adenoma, 2 cases of adrenal cyst, 2 cases of teratoma, 1 case of pheochromocytoma, 1 case of nerve sheath tumor, and 1 case of adrenal hemorrhage; and 20 cases of malignant tumors, including 10 cases of neuroblastoma, 9 cases of ganglioneuroblastoma, and 1 case of adrenocortical carcinoma.

**Conclusions:**

Neuroblastoma is the most common type of nonneonatal AI, and detailed laboratory investigations and imaging studies are recommended for aggressive evaluation and treatment in this population. The rate of spontaneous regression of AI is high in neonates, and close observation is feasible if the tumor is small, confined to the adrenal gland and has no distant metastasis.

## Background

The incidence of adrenal incidentaloma (AI) is increasing due to the increased frequency of imaging and improved imaging sensitivity [1]. AI is relatively common in adults, and several organizations, such as the American Association of Clinical Endocrinologists/American Association of Endocrine Surgeons and the European Society Endocrinology, have proposed specific protocols to guide the evaluation, treatment, and follow-up management of AI in adults [2]. Although AI, a nonfunctioning adrenocortical adenoma, is most common in adults, neuroblastoma is the most common incidental tumor of the adrenal gland in children. In addition, in the neonatal period, which is a more complex stage of childhood, the biology of adrenal masses found in this age group is also more specific, and the nature of these masses can range from spontaneous regression to rapid progression to aggressive disease with metastatic dissemination and even death. Given that AI is the most common malignant tumor, the management of AI in children cannot be simply based on the measurements used in adult AI. In this study, we retrospectively analyzed the clinical data of pediatric AI patients in a single center to investigate the clinical characteristics and management of AI in children.

## Methods

A total of 66 children with adrenal tumors were diagnosed and treated at the Department of Urology of the Children’s Hospital of Nanjing Medical University from December 2016 to December 2022. A total of 55 cases were detected during physical examination, or the patients were diagnosed and received treatment for diseases other than adrenal disease after excluding adrenal tumors detected due to typical clinical manifestations or signs such as centripetal obesity and precocious puberty. Research protocols involving human materials were approved by the Medical Ethics Committee of the Children’s Hospital of Nanjing Medical University. All clinical information, radiological diagnosis, laboratory test results, intervention results, and follow-up data were collected from the department’s database.

All the children underwent ultrasonography and CT scanning, and 11 children underwent MRI. In addition to routine tests such as blood routine and biochemical indexes, the examination and evaluation of adrenal endocrine hormones and tumor markers included (1) plasma cortisol and ACTH levels, (2) plasma catecholamine and metabolite determination, (3) plasma renin and plasma aldosterone, (4) urinary vanillylmandelic acid/homovanillic acid(VMA/HVA), and (5) AFP, CEA, NSE, and CA19-9. Five patients underwent a low-dose dexamethasone suppression test. Seventeen of the 55 patients were treated with watch-waiting therapy, 4 of the 17 ultimately underwent surgery, 4 of the 38 patients underwent tumor biopsy, and 34 underwent adrenalectomy.

The data were analyzed using Graph Pad Prism 8. The measurement data are expressed as ‾*x* ± *sd*. The maximum diameter of the tumors, age of the patients with benign and malignant tumors, and maximum diameter of the tumors between the laparoscopic surgery group and the open surgery group were compared using paired t tests, and the percentages of the count data were compared using Fisher’s exact test.

## Results

In this study, all patients were divided into two groups according to their age at the time of consultation: the neonate group and the nonneonate group.

Neonate group:

There were 7 male and 10 female patients, 7 of whom were diagnosed via prenatal examination and 10 of whom were diagnosed after birth. Five patients were diagnosed with lesions on the left side, 12 patients were diagnosed with lesions on the right side, and the maximal diameters of the masses ranged from 16 to 48 mm. The characteristics of the AIs in the neonate group are presented in Table 1.

**Table 1:** Characteristics of AI in the neonates group

Characteristic		n (%)
Sex	Boy	7(41.2)
	Girl	10(58.8)
Site	Left	5(29.4)
	Right	12(70.6)
Discovery Time	Prenatal	7(41.2)
	Postnatal	10(58.8)
Size	˂3.1 cm	6(35.3)
	≥3.1 cm	11(64.7)
	˂5 cm	17(100)
	≥5 cm	0(0)
CT description	Cystic	8(47.1)
	Solid-cystic	5(29.4)
	Solid	4(23.5)
Intervention	Observation	13(76.5)
	Surgical treatment	4(23.5)

Among the 17 patients, 8 had cystic masses with a maximum diameter of 16$\sim$48 mm, 5 had cystic-solid masses with a maximum diameter of 33$\sim$39 mm, and 4 had solid masses with a maximum diameter of 18$\sim$45 mm. Two patients with solid adrenal gland masses suggested by CT scan had obvious elevations in serum NSE and maximum diameters of 44 and 45 mm, respectively. These patients underwent adrenal tumor resection, and the pathology diagnosed that they had neuroblastomas(NB). In one patient, the right adrenal gland was 26 × 24 × 27 mm in size with slightly elevated echogenicity at 38 weeks after delivery, and the mass increased to a size of 40 × 39 × 29 mm according to the 1-month postnatal review. MRI suggested that the adrenal gland tumor was associated with liver metastasis, and the pathology of the tumor suggested that it was NB associated with liver metastasis after surgical resection (stage 4 S, FH). One child was found to have 25 × 24 × 14 mm cystic echoes in the left adrenal region during an obstetric examination, and ultrasound revealed 18 × 11 mm cystic solid echoes 5 days after birth. Ultrasound revealed 24 × 15 mm cystic solid echoes at 2 months. Serum NSE and urinary VMA were normal, and the tumor was excised due to the request of the parents. Pathology suggested a teratoma in the postoperative period. A total of 13 children did not receive surgical treatment or regular review via ultrasound, serum NSE or urine VMA. The follow-up time ranged from 1 to 31 months, with a mean of 9.04 ± 7.61 months. Eight patients had complete swelling, and 5 patients were significantly younger than the previous patients. Nonneonate group:

There were 24 male and 14 female patients in the nonneonate group; 24 patients had lesions on the left side, 14 patients had lesions on the right side, and the maximal diameters of the masses ranged from 17 to 131 mm. Most of these tumors were found during routine physical examinations or incidentally during examinations performed for various complaints, such as gastrointestinal symptoms, respiratory symptoms, or other related conditions. As shown in Table 2, abdominal pain was the most common risk factor (44.7%) for clinical onset, followed by routine physical examination and examination for respiratory symptoms.

**Table 2:** Clinical presentations leading to discovery of AI in non-neonate group

Clinical presentation	n (%)
Abdominal pain	17(44.7)
Medical checkup	7(18.4)
Fever	4(10.5)
Pneumonia	3(7.9)
Cough	1(2.6)
Diarrhea	1(2.6)
Vomiting	1(2.6)
Urinary tract infection	1(2.6)
Low back pain	1(2.6)
Hodgkin’s lymphoma	1(2.6)
Vaginal bleeding	1(2.6)

Among the 38 patients, 10 had NBs with maximum diameters ranging from 20 to 131 mm, 9 had ganglion cell neuroblastomas with maximum diameters ranging from 33.6 to 92 mm, 9 had ganglion cell neuromas with maximum diameters ranging from 33 to 62 mm, 2 had adrenal adenomas with maximum diameters ranging from 17 to 70 mm, 1 had a cortical carcinoma with a maximum diameter of 72 mm, 2 had adrenal cysts with maximum diameters ranging from 26 to 29 mm, 2 had mature teratomas with maximum diameters of 34 and 40 mm, 1 had a pheochromocytoma with a diameter of 29 mm, 1 had a nerve sheath tumor with a diameter of 29 mm, and 1 patient with postoperative pathological confirmation of partial hemorrhagic necrosis of the adrenal gland had focal calcification with a maximum diameter of 25 mm (Table 3).

**Table 3:** Distribution of different pathologies among AI with various sizes in non-neonate group

Clinical diagnosis	No.	Size <40 mm	Size 40$\sim$60mm	Size >60 mm
Neuroblastoma	10	1	4	5
Ganglioneuroblastoma	9	3	3	3
Ganglioneuromas	9	0	8	1
Adrenocortical adenoma	2	1	0	1
Adrenocortical carcinoma	1	0	0	1
Cyst	2	2	0	0
Mature teratoma	2	0	2	0
Pheochromocytoma	1	1	0	0
Nerve sheath tumor	1	1	0	0
Hematoma	1	1	0	0

The mean age of children with malignant tumors was significantly lower than that of children with benign tumors (57.95 ± 37.20 months vs. 105.0 ± 23.85 months; *t* = 4.582, *P* < 0.0001). The maximum diameter of malignant tumors ranged from 20 to 131 mm, while that of benign tumors ranged from 17 to 72 mm, and the maximum diameter of malignant tumors was significantly greater than that of benign tumors (65.15 ± 27.61 mm v 37.59 ± 12.98 mm; *t* = 3.863, *P* = 0.0004). Four biopsies, 5 laparoscopic adrenal tumor resections and 11 open adrenal tumor resections were performed for malignant tumors, and 16 laparoscopic adrenal tumor resections and 2 open procedures were performed for benign tumors. The maximum diameter of the tumors ranged from 17 to 62 mm in 21 children who underwent laparoscopic surgery and from 34 to 99 mm in 13 children who underwent open resection; there was a statistically significant difference in the maximum diameter of the tumors between the laparoscopic surgery group and the open surgery group (35.63 ± 10.36 mm v 66.42 ± 20.60 mm; *t* = 5.798, *P* < 0.0001).

Of the 42 children with definitive pathologic diagnoses at surgery, 23 had malignant tumors, and 19 had benign tumors. There were 15 malignant tumors with calcification on imaging and 5 benign tumors. The percentage of malignant tumors with calcifications in was significantly greater than that of benign tumors (65.22% v 26.32%; *P* = 0.0157). In the present study, all the children underwent CT, and 31 patients had postoperative pathological confirmation of NB. A total of 26 patients were considered to have neurogenic tumors according to preoperative CT, for a diagnostic compliance rate of 83.97%. Three children were considered to have cortical adenomas by preoperative CT, and 1 was ultimately diagnosed by postoperative pathology, for a diagnostic compliance rate of 33.33%. For 4 patients with teratomas and adrenal cysts, the CT findings were consistent with the postoperative pathology. According to our research, NB 9-110HU, GNB 15-39HU, GB 19-38HU, ACA 8HU, adrenal cyst 8HU, and cellular achwannoma 17HU.

## Discussion

According to the clinical practice guidelines developed by the European Society of Endocrinology and European Network for the Study of Adrenal Tumors, AI is an adrenal mass incidentally detected on imaging not performed for a suspected adrenal disease [3]. The prevalence of AI is approximately 4%, and the incidence increases with age [4]. Most adult AIs are nonfunctioning benign adrenal adenomas (up to 75%), while others include functioning adrenal adenomas, pheochromocytomas, and adrenocortical carcinomas [5]. In contrast to the disease spectrum of adult AI cases, NB is the most common tumor type among children with AI, and benign cortical adenomas, which account for the vast majority of adult AI, accounting for less than 0.5% of cases in children [6]. According to several guidelines, urgent assessment of an AI is recommended in children because of a greater likelihood of malignancy [3, 7].

When an adult patient is initially diagnosed with AI, it should be clear whether the lesion is malignant and functional. In several studies, the use of noncontrast CT has been recommended as the initial imaging method for adrenal incidentaloma; a CT attenuation value ≤ 10 HU is used as the diagnostic criterion for benign adenomas; and these methods have a specificity of 71-79% and a sensitivity of 96-98% [8, 9]. A CT scan of tumors with diameters greater than 4 to 6 cm, irregular margins or heterogeneity, a CT attenuation value greater than 10 HU, or a relative contrast enhancement washout of less than 40% 10 or 15 min after administration of contrast media on enhanced CT is considered to indicate potential malignancy [7]. As the most common AI in children, NB often appears as a soft tissue mass with uneven density on CT, often accompanied by high-density calcified shadows, low-density cystic lesions or necrotic areas. CT scans can easily identify more typical NBs, and for those AIs that do not show typical calcified shadows on CT, it is sometimes difficult to differentiate neurogenic tumors from adenomas. In these patients, except for the 1 patient with adrenal cysts who had a CT value of 8 HU, very few of the remaining AI patients had a CT value less than 10 HU. Therefore, the CT value cannot be used simply as a criterion for determining the benign or malignant nature of AI, and additional imaging examinations, such as CT enhancement, MRI, and FDG-PET if necessary, should be performed immediately for AI in children.

Initial hormonal testing is also needed for functional assessment, and aldosterone secretion should also be assessed when the patient is hypertensive or hypokalemic [7]. Patients with AI who are not suitable for surgery should be observed during the follow-up period, and if abnormal adrenal secretion is detected or suggestive of malignancy during this period, prompt adrenal tumor resection is needed. For adult patients with AI, laparoscopic adrenal tumor resection is one of the most effective treatments that has comparative advantages in terms of hospitalization time and postoperative recovery speed; however, there is still some controversy over whether to perform laparoscopic surgery for some malignant tumors with large diameters, especially adrenocortical carcinomas, and some studies have shown that patients who undergo laparoscopic surgery are more prone to peritoneal seeding of tumors [10].

The maximum diameter of an adult AI is a predictor of malignancy, and a study by the National Italian Study Group on Adrenal Tumors, which included 887 AIs, showed that adrenocortical carcinoma was significantly correlated with the size of the mass, and the sensitivity of detecting adrenocortical carcinoma with a threshold of 4 cm was 93% [11]. According to the National Institutes of Health, patients with tumors larger than 6 cm should undergo surgical treatment, while patients with tumors smaller than 4 cm should closely monitored; for patients with tumors between 4 and 6 cm, the choice of whether to be monitored or surgically treated can be based on other indicators, such as imaging [12]. A diameter of 4 cm is not the initial threshold for determining the benign or malignant nature of a mass in children.

In a study of 26 children with AI, Masiakos et al. reported that 9 of 18 benign lesions had a maximal diameter less than 5 cm, 4 of 8 malignant lesions had a maximal diameters less than 5 cm, and 2 had a diameter less than 3 cm. The mean maximal diameter of benign lesions was 4.2 ± 1.7 cm, whereas the mean maximum diameter of malignant lesions was 5.1 ± 2.3 cm. There was no statistically significant difference between the two comparisons; therefore, this study concluded that children with AI diameters less than 5 cm cannot be treated expectantly [6]. Additionally, this study revealed that malignant lesions occurred significantly more frequently than benign lesions in younger children (mean age 1.7 ± 1.8 years v 7.8 ± 5.9 years; *P* = 0.02).

In the nonneonatal group of this study, 20 patients with malignant tumors had maximum diameters ranging from 20 to 131 mm, 10 had malignant tumors larger than 60 mm, and 3 had tumors smaller than 40 cm; 18 patients with benign tumors had maximum diameters ranging from 17 to 70 mm, 5 had diameters ranging from 40 to 60 mm, and 5 had diameters larger than 60 mm. Therefore, it is not recommended to use the size of the largest diameter of the tumor to decide whether to wait and observe or intervene surgically for children with AI. Instead, it is necessary to consider the age of the child; laboratory test results, such as whether the tumor indices are elevated or not; whether the tumor has an endocrine function; etc.; and imaging test results to make comprehensive judgments and decisions. Preoperative aggressive evaluation and prompt surgical treatment are recommended for nonneonatal pediatric AI patients.

Adrenal hematoma and NBs are the most common types of adrenal area masses in children, while pheochromocytoma, adrenal cyst, and teratoma are rarer masses [13]. In clinical practice, adrenal hematoma and NB are sometimes difficult to differentiate, especially when adrenal masses are found during the prenatal examination and neonatal period, and such children need to be managed with caution. The Children’s Oncology Group (COG ANBL00B1) implemented the watchful waiting treatment for children under 6 months of age with a solid adrenal mass < 3.1 cm in diameter (or a cystic mass < 5 cm) without evidence of distant metastasis, and if there is a > 50% increase in the adrenal mass volume, there is no return to the baseline VMA or HVA levels, or if there is a > 50% increase in the urinary VMA/HVA ratio or an inversion, surgical resection should be performed [14]. Eighty-three children in this study underwent expectant observation, 16 of whom ultimately underwent surgical resection (8 with INSS stage 1 NB, 1 with INSS stage 2B, 1 with INSS stage 4 S, 2 with low-grade adrenocortical neoplasm, 2 with adrenal hemorrhage, and 2 with extralobar pulmonary sequestration). Most of the children who were observed had a reduced adrenal mass volume. Of the 56 patients who completed the final 90 weeks of expectant observation, 27 (48%) had no residual mass, 13 (23%) had a residual mass volume of 0–1 ml, 8 (14%) had a mass volume of 1–2 ml, and 8 (14%) had a volume of > 2 ml; ultimately, 71% of the residual masses had a volume ≤ 1 ml and 86% had a residual volume ≤ 2 ml. In this study, a total of 16 patients were included in the watchful waiting treatment group; 3 patients underwent surgical treatment during the follow-up period, and 13 patients ultimately completed watchful waiting treatment. After 1–31 months of follow-up, 8 patients’ swelling completely disappeared, and 5 patients’ swelling significantly decreased. After strict screening for indications and thorough follow-up review, AIs in the neonatal period can be subjected to watchful waiting treatment, and satisfactory results can be achieved.

For benign adrenal tumors, laparoscopic surgery is superior to open surgery in terms of successful resection, whereas the feasibility of minimally invasive surgery for AI with preoperative suspicion of malignancy is controversial. The European Cooperative Study Group for Pediatric Rare Tumors recommends that minimally invasive surgery be considered only for early childhood tumors and should be limited to small, localized tumors; additionally, imaging should suggest no invasion of surrounding tissue structures or lymph nodes; and this strategy requires surgeons with extensive experience in oncologic and adrenal surgery [15]. NB is the most common pediatric AI, and open tumor resection remains the mainstay of treatment. For small, early tumors without evidence of invasion on preoperative examination, laparoscopic resection may be considered if the principles of oncologic surgery can be adhered to. If the patient responds to chemotherapy, the decision to perform laparoscopic tumor resection can also be re-evaluated after chemotherapy. According to the current study, the recurrence and mortality rates of laparoscopic surgery are comparable to those of open surgery [16, 17]. The relative contraindications for laparoscopic NB resection include a tumor diameter greater than 6 cm, venous dilatation, and the involvement of adjacent organs or blood vessels [18]. Patients who undergo open adrenalectomy have higher overall survival and recurrence-free survival rates than patients who undergo laparoscopic adrenalectomy [19]. Open adrenalectomy remains the gold standard for surgical resection of adrenocortical carcinoma, whereas laparoscopic adrenalectomy should be reserved for highly selected patients and performed by surgeons with appropriate expertise [20].

Cortical tumors are particularly rare among children with AIs and are sometimes not clearly distinguishable from neurogenic tumors on preoperative imaging; in such patients, the presence of subclinical Cushing’s syndrome needs to be carefully evaluated preoperatively; otherwise, a perioperative adrenal crisis may occur [21]. In patients in whom the possibility of an adrenocortical tumor was considered preoperatively, the assessment for subclinical Cushing’s syndrome mainly involved assessing the serum dehydroepiandrosterone sulfate level and performing an overnight dexamethasone suppression test.

**Fig. 1:**
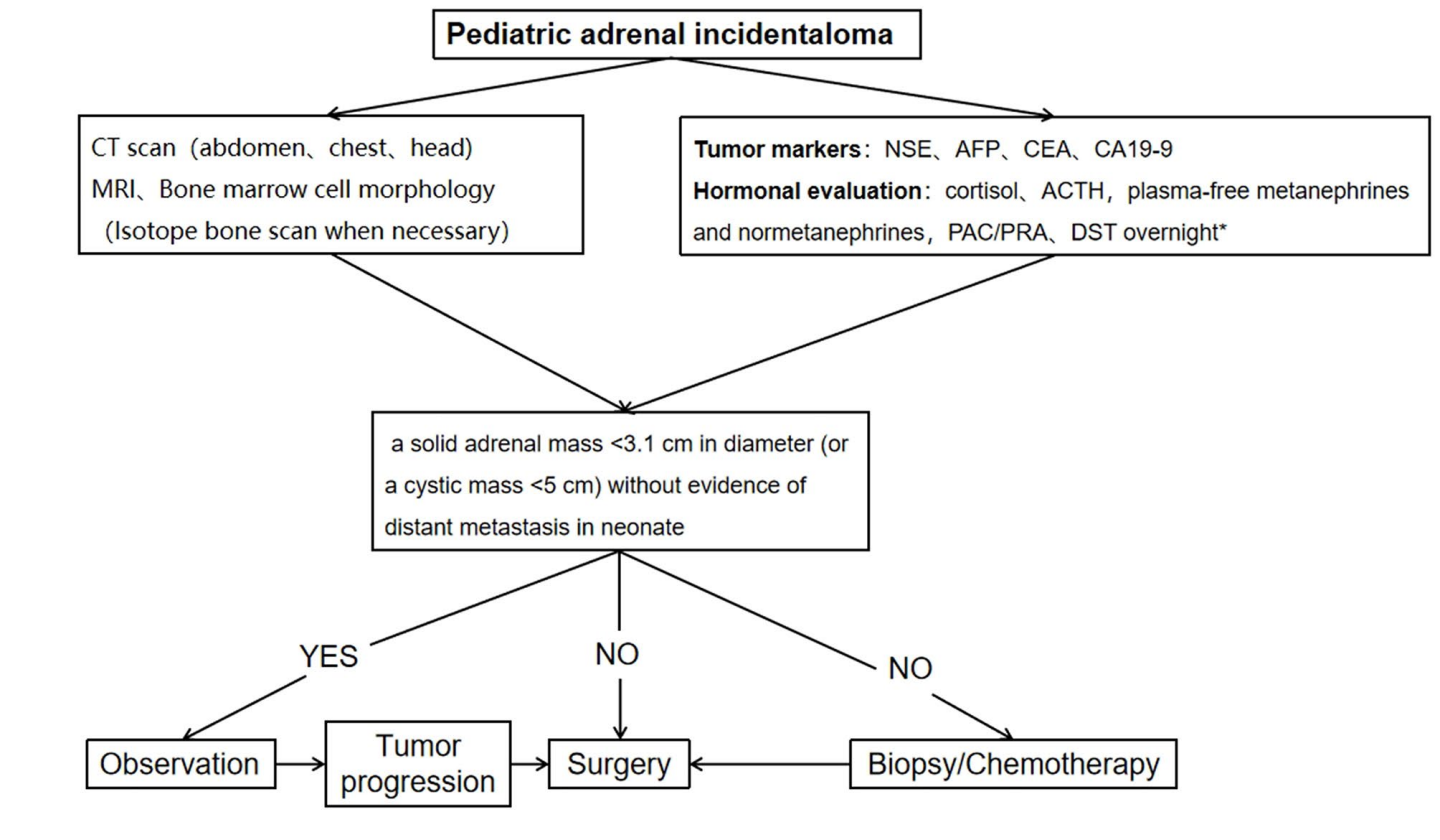
Algorithm for the evaluation and management of a pediatric adrenal incidentaloma. *DST overnight :20µg/kg dexamethasoneweight ˂40 kg,1 mg dexamethasone if ≥ 40 kg. CT = computed tomographic;MRI = magnetic resonance imaging;NSE = neuron-specific enolase;AFP = alpha-fetoprotein;CEA = carcinoembryonic antigen;CA19-9 = cancerantigen19-9;ACTH = adrenocorticotropic hormone;PAC = plasma aldosterone concentration; PRA = plasma renin activity;DST = dexamethasone suppression test

A procedure for evaluating pediatric AI is shown in Fig. 1. Imaging is the first step in the evaluation of AI in children. CT can be used to clarify the nature of most tumors. MRI can be used to evaluate imaging risk factors (IDRFs) for NB. Bone marrow cytomorphology is recommended for all children with AI, along with microscopic residual neuroblastoma testing and further bone scanning if the bone marrow examination is positive. In addition, serum tumor marker levels and other relevant tests should be performed, and hormone levels should be evaluated. If adrenal adenomas cannot be completely excluded during the preoperative examination, a 1 mg overnight dexamethasone suppression test should be performed to exclude subclinical Cushing’s syndrome. In patients with hypertensive hypokalemia, the presence of aldosteronism should be evaluated by testing plasma aldosterone concentrations and plasma renin activity. Adrenal masses found in the neonatal period can be observed if the tumor is small, confined to the adrenal gland and shows no evidence of distant metastasis, while tumors that increase significantly in size during the follow-up period or that are associated with persistently elevated tumor markers require aggressive surgical treatment.

## Data Availability

The datasets analyzed during the current study are not public, but are available from the corresponding author on reasonable request.

## Abbreviations

CT: computed tomographic
MRI: magnetic resonance imaging
ACTH: adrenocorticotropic hormone
VMA: vanillylmandelic acid
HVA: homovanillic Acid
AFP: alpha-fetoprotein
CEA: carcinoembryonic antigen
NSE: neuron-specific enolase
CA19-9: cancerantigen19-9
FH: favorable histology
HU: Hounsfiled Unit
COG: Children’s Oncology Group
INSS: International Neuroblastoma Staging System
